# Reactivation of Pulmonary Tuberculosis During Treatment of Chronic Myelomonocytic Leukemia

**DOI:** 10.7759/cureus.15491

**Published:** 2021-06-07

**Authors:** Jay Pescatore, Ashley Cohen, Krishna Moturi, Ruben Hernandez-Acosta

**Affiliations:** 1 Medicine, John H. Stroger, Jr. Hospital of Cook County, Chicago, USA; 2 Emergency Medicine, University of Michigan, Ann Arbor, USA

**Keywords:** active pulmonary tuberculosis, chronic myelomonocytic leukemia, latent tuberculosis infection, tuberculosis/ transmission, tuberculosis testing, microbiology, infection microbiology

## Abstract

A 76-year-old woman from a tuberculosis (TB) endemic region with chronic myelomonocytic leukemia (CMML) on Azacitidine presented with a non-productive cough. A CT scan of the chest revealed a lobulated opacity in the right upper lobe and antibiotic therapy was initiated for a potential bacterial pneumonia. However, a high suspicion for pulmonary TB remained given her nation of origin, immunosuppression, and imaging findings. Three sputum and bronchoalveolar lavage (BAL) acid-fast bacilli (AFB) smears with PCR testing for *Mycobacterium tuberculosis* were negative, as were examinations for other potential fungal or bacterial etiologies of the patient’s symptoms and imaging findings. While awaiting final TB culture results from BAL, her CMML underwent a transformation to acute myeloid leukemia (AML). Given the urgent need for initiation of chemotherapy, empiric treatment for TB was commenced while awaiting the final TB culture. Within 48-hours of initiating therapy for TB, the patient’s fevers subsided. One week after discharge our team was notified of a positive *M. tuberculosis* culture from BAL. We suspect that our patient had a latent TB infection which reactivated due to her CMML. This case highlights the importance of maintaining a high clinical suspicion for TB in high-risk patients, even in the case of initially negative laboratory examinations. Further, it demonstrates the importance of screening and treating latent TB in patients with leukemias.

## Introduction

Active tuberculosis (TB) most often results from the reactivation of a prior latent infection. Populations of individuals including those with HIV, solid organ transplant history, chronic renal failure, or diabetes have been identified by both the Centers for Disease Control (CDC) and American Thoracic Society (ATS) as at high risk for developing active TB [1].

This case highlights the importance of awareness of the increased risk for reactivation TB in patients with myelodysplastic syndrome/myeloproliferative disorder (MDS/MPD), particularly those from TB-endemic regions. It also demonstrates the importance of screening and treatment of latent TB in patients with leukemia. Further, it reinforces the importance of clinical judgement in cases where confirmatory exam results may take prolonged periods of time to result.

This case report has been presented at the 2020 national CHEST conference (https://journal.chestnet.org/article/S0012-3692(20)32659-3/fulltext). 

## Case presentation

A 76-year-old woman with a four-month history of chronic myelomonocytic leukemia (CMML) being treated with Azacitidine presented to the emergency department with a chief complaint of a non-productive cough for one month. She endorsed 10 days of recurrent fevers, pleuritic chest pain, and night sweats. She also reported a 10 lb weight loss which she had attributed to a decreased appetite from chemotherapy. She denied any recent travel history but did mention that her husband was treated for latent TB 10 years prior.

**Figure 1:**
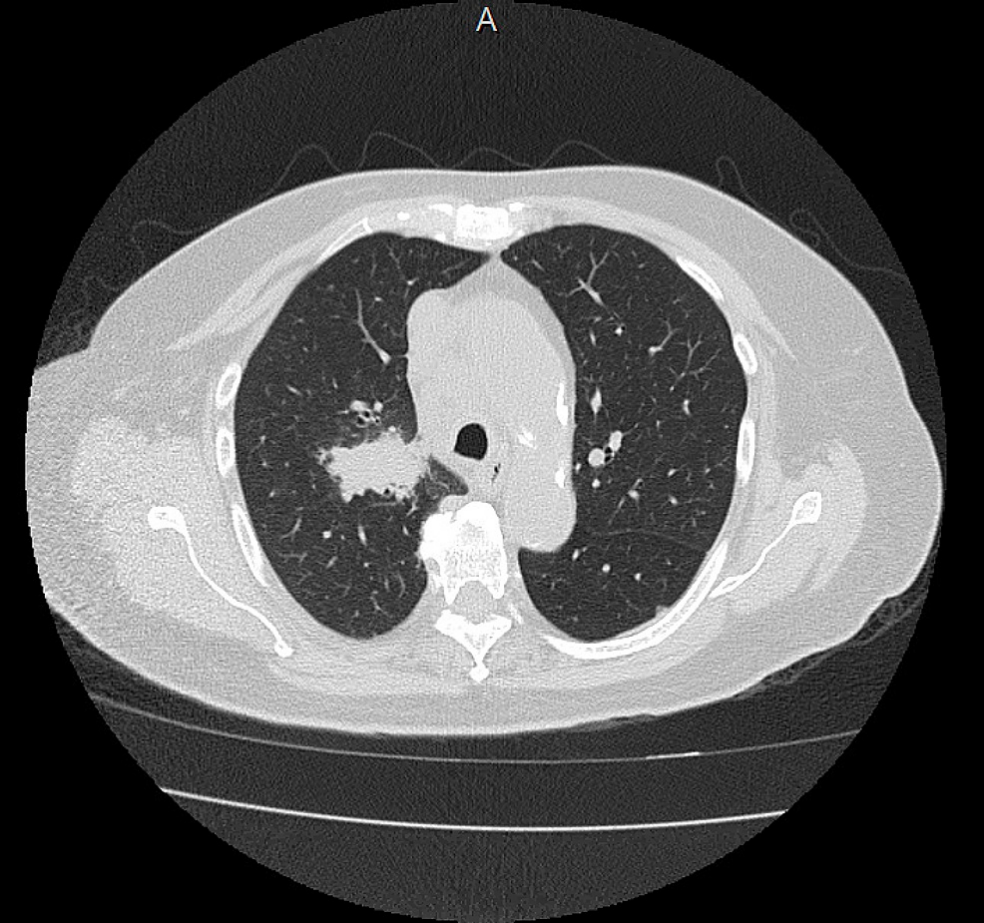
Cross-sectional CT Chest of Right Upper Lobe Opacity

On the initial examination, the patient was tachycardic, afebrile, and otherwise hemodynamically stable. Other than a mild leukocytosis to 12.5 k/µL, laboratory examinations were unremarkable. A chest CT scan revealed a new lobulated opacity of the right upper lobe as well as new mediastinal, supraclavicular, and upper abdominal lymphadenopathy (Figure 1). This was as compared to CT imaging performed at the time of her initial CMML diagnosis four months earlier.

The patient was admitted and empirically started on vancomycin and meropenem for hospital-acquired pneumonia. Given her history of TB exposure, current immunosuppression, and the radiologic findings, a high suspicion for TB remained. However, early in her hospitalization, three acid-fast bacilli (AFB) smears with nucleic-acid amplification testing (NAAT-PCR) for *Mycobacterium tuberculosis* were negative. Throughout the hospital course and despite treatment with broad-spectrum antibiotics the patient continued to have a persistent fever with an oral temperature maximum of 38.7 °C and at least one reading of 37.9 °C or higher daily during the first eight days of admission. This prompted further infectious diseases work-up, with a particular focus on fungal infections which might have mimicked the presentation of TB. Such examinations included serum galactomannan, beta-D-glucan, cryptococcal antigen, urinary Legionella, Blastomyces, and Histoplasma antigens, all of which were negative. A QuantiFERON Gold (IFN-gamma release assay) was positive. Given the patient’s continued symptoms, negative infectious diseases work-up, and unresponsiveness to antibiotic therapy, TB was still suspected. The patient continued to have only scant sputum production despite induction attempts; therefore, a bronchoscopy was planned in order to obtain bronchoalveolar lavage (BAL) in hopes of better targeting her therapies. BAL from the patient’s right upper lobe tested negative for fungal antigens and were culture-negative for bacteria or fungi. The AFB smear and PCR from the BAL were also negative, but formal BAL TB cultures were ordered.

## Discussion

Patients with hematologic malignancies are considered a high-risk group for having active TB, most notably lymphoma and myelodysplastic syndrome/myeloproliferative disorder (MDS/MPN) [2]. In a large population-based cohort study patients with MDS, MPN, or lymphoma were found to have the greatest risk for TB in comparison to patients with other malignancies [2]. This increased susceptibility to TB is either a direct consequence of immunosuppression from the malignancy, secondary to therapy, or a combination of both. The malignancy-related decrease in T cells, especially CD4 type, directly results in immunosuppression. Therapy with corticosteroids, fludarabine, or hematopoietic stem cell transplantation may also play a significant role in further suppression of T cell function [3]. Case reports of platinum-based chemotherapeutic agents and radiation therapy have also been reported to cause immunosuppression allowing for reactivation of TB [4].

We suspect that our patient from a TB-endemic region with a known exposure to latent TB had latent TB infection herself which was subsequently reactivated. We propose that the reactivation in this patient was primarily the result of her CMML, classified as an overlap of MDS/MPN. It is also possible that the patient’s treatment with the antimetabolite therapy Azacitidine, through further immunosuppression and dampening of T-cell responses, contributed to her risk for reactivation as well. Although Azacitidine has been reported to cause pneumonitis [5], no incidents of TB have been reported in prior case reviews [6,7]. It is possible, however, that Azacitidine, in causing a dose-dependent loss of T-cell populations, could increase the risk for reactivation of TB [8].

## Conclusions

In this case, a high index of clinical suspicion for tuberculosis, despite negative AFB smears and NAAT-PCR, allowed for the timely treatment of the patient. This case highlights the importance of an awareness of the increased risk for TB reactivation in patients with MDS/MPN, particularly those from TB-endemic regions. It also demonstrates the importance of screening for and treatment of latent TB in patients with leukemias. Further, it reinforces the importance of clinical judgement in cases where confirmatory examination results may take prolonged periods of time.
